# Changes in perioperative serum transaminase levels: predicting early recurrence after hepatectomy for hepatocellular carcinoma

**DOI:** 10.3389/fonc.2025.1589884

**Published:** 2025-05-19

**Authors:** Yingfei Wei, Guixiang Qian, Tao Meng, Zhong Tong

**Affiliations:** Department of Hepatobiliary and Pancreatic Surgery, The Third Affiliated Hospital, Anhui Medical University, Hefei, China

**Keywords:** hepatocellular carcinoma, hepatectomy, transaminases, early recurrence, predictive model

## Abstract

**Background and purpose:**

Hepatocellular carcinoma (HCC) is associated with poor prognosis due to its high propensity for early postoperative recurrence. In this study, we aimed to develop a novel model based on changes in perioperative aspartate aminotransferase (AST) and alanine aminotransferase (ALT) levels to predict early recurrence following hepatectomy for HCC.

**Methods:**

This study is a dual-center retrospective cohort study. Based on strict inclusion and exclusion criteria, 317 hepatocellular carcinoma (HCC) patients from Center 1 and 58 patients from Center 2 were enrolled. Patients from Center 1 were randomly allocated in a 7:3 ratio into a training set (n=221) and an internal validation set (n=96), while Center 2 served as an independent external validation set. In the training set, independent risk factors associated with early recurrence after hepatectomy for HCC were identified through univariate and multivariate analyses, and a predictive model was constructed. The predictive performance was evaluated using the area under the receiver operating characteristic (ROC) curve (AUC). Calibration curves and decision curve analysis (DCA) were employed to assess model calibration and clinical utility, respectively. Additionally, model interpretability was visualized through the SHapley Additive exPlanations (SHAP) framework. Based on the combined model’s predictions, this study further stratified patients’ two-year progression-free survival (PFS) and five-year overall survival (OS) using Kaplan-Meier curves.

**Results:**

Univariate and multivariate analyses revealed that alpha-fetoprotein (AFP), total bilirubin (TB), postoperative ALT (ALTp), HBV infection history, tumor size, and change in AST and ALT (CAA) were independent risk factors for early recurrence (P<0.05). The predictive model incorporating these factors achieved an AUC of 0.804, demonstrating robust predictive capability. The model exhibited strong consistency between predicted outcomes and actual observations in the training, internal validation, and external validation sets.

**Conclusion:**

This retrospective cohort study successfully established a predictive model for early recurrence after hepatectomy in HCC patients, highlighting its potential clinical utility.

## 1 Introduction

Hepatocellular Carcinoma (HCC) is one of the malignancies with high global incidence and mortality rates. According to the 2020 Global Cancer Statistics, HCC ranks as the sixth most common cancer worldwide, with approximately 906,000 new cases annually, accounting for 4.7% of all cancer cases. Simultaneously, HCC is the third leading cause of cancer-related deaths, with about 830,000 annual deaths, representing 8.3% of all cancer-related fatalities (1). China has the highest number of HCC cases globally, contributing to approximately 45%-50% of the global HCC burden. According to the latest data from the National Cancer Center of China, there were about 410,000 new HCC cases and 390,000 deaths in China in 2022 (2). In recent years, with advancements in ablation therapy and intravascular interventional therapy, treatment options for HCC have become more comprehensive. However, hepatectomy remains the primary treatment modality for HCC patients. Despite surgical resection, HCC has a high likelihood of recurrence (3). Early recurrence of HCC is typically defined as tumor recurrence within 24 months after curative treatment (surgical resection or ablation), as opposed to late recurrence (>24 months) (4). Recent studies report that the probability of early recurrence ranges between 25% and 50% (5). Early recurrence significantly impacts patient prognosis: 1) It leads to a sharp decline in survival rates, with a median survival of only 8–15 months after recurrence (6); 2) It limits treatment options, as only 20%-30% of patients are suitable for secondary curative treatment due to reduced liver function reserve and multifocal tumors (7); 3) It accelerates liver failure, particularly in patients with underlying cirrhosis (8); 4) It increases financial burden and psychological stress due to higher treatment costs post-recurrence. Therefore, effective early prediction and intervention strategies are crucial for HCC patients at high risk of recurrence. Early prediction can provide timely treatment decisions, improve survival rates, prolong survival, and significantly enhance patients’ quality of life.

During liver resection surgery, a low central venous pressure (CVP) strategy is often employed to reduce hepatic blood flow, which inevitably leads to hepatic ischemia/reperfusion injury (9–11). Postoperative liver function impairment may accelerate tumor recurrence, and changes in serum transaminase levels are the most direct indicators reflecting alterations in liver function. CAA, or the change in aspartate aminotransferase (AST) and alanine aminotransferase (ALT) levels between postoperative day 3 and preoperative values, is calculated using a fusion index formula based on the Euclidean norm. Wang et al., in a recent issue of the “Annals of Surgery”, proposed the CAA scoring model to predict postoperative complications (12). Lu et al. further evaluated long-term survival outcomes after hepatectomy by studying changes in serum transaminases (13). In previous research, Kostakis et al. (14)used preoperative ALT and AST changes to predict patient prognosis. However, no studies have yet explored the changes in preoperative and postoperative serum transaminases to predict early recurrence in HCC patients after hepatectomy.

Several methods currently exist to predict early recurrence in HCC patients after hepatectomy, such as the Singapore Liver Cancer Recurrence Score (15) and the Italian Liver Cancer Program Score (16). While these scoring systems demonstrate good predictive performance, they have limitations, including a lack of extensive clinical studies and external validation. The albumin-bilirubin (ALBI) score model proposed by Lee et al. (17)shows excellent performance in predicting overall survival (OS) in HCC patients after hepatectomy, outperforming the Child-Pugh classification. However, research on recurrence-free survival remains limited. This study aims to further investigate the changes in preoperative and postoperative serum transaminases to predict early recurrence in HCC patients after hepatectomy, providing theoretical support for clinicians to develop subsequent treatment plans and improve patients’ quality of life.

## 2 Patients and methods

This study is a dual-center retrospective investigation approved by the Ethics Committees of the First Affiliated Hospital of the University of Science and Technology of China (Anhui Provincial Hospital) and the Third Affiliated Hospital of Anhui Medical University (Hefei First People’s Hospital). Due to the retrospective nature of the study, patient informed consent was waived. The research was conducted in strict accordance with the ethical guidelines of the 1975 Declaration of Helsinki. The First Affiliated Hospital of University of Science and Technology of China (Anhui Provincial Hospital) Ethics Research Approval:2024-KY585,The Third Affiliated Hospital of Anhui Medical University (Hefei First People’s Hospital) Ethics Research Approval: NO.2025-031-01.

**Figure 1:**
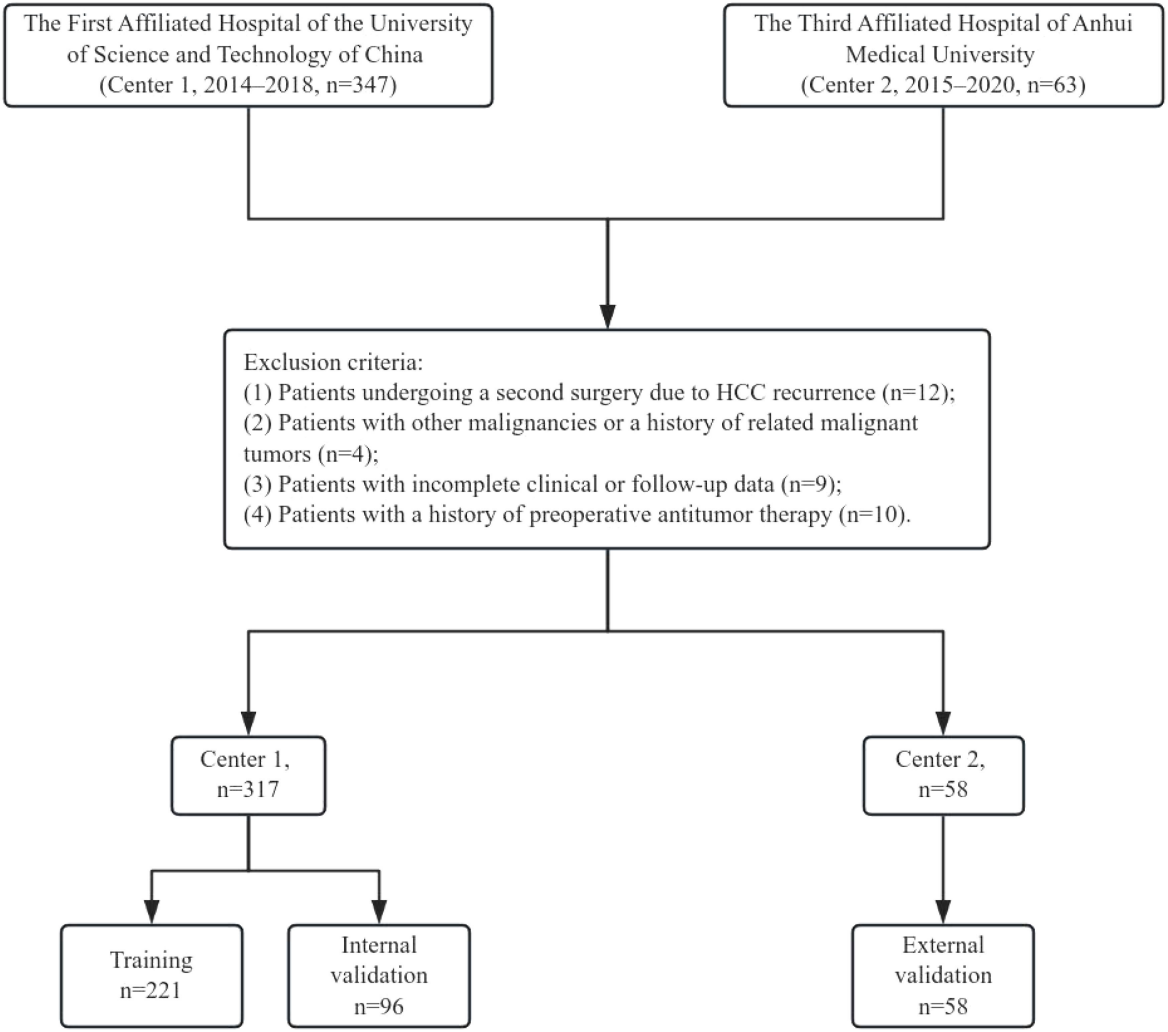
Flowchart.

A total of 347 HCC patients from the First Affiliated Hospital of the University of Science and Technology of China (Anhui Provincial Hospital) (Center 1) between 2014 and 2018, and 63 HCC patients from the Third Affiliated Hospital of Anhui Medical University (Hefei First People’s Hospital) (Center 2) between 2015 and 2020 were included in this study. The inclusion criteria were as follows: (1) patients aged 18 years or older who underwent their first hepatectomy at the respective hospital;(2) Patients with BCLC stage 0 and A (The possibility of major vascular invasion is ruled out by enhanced CT);(3) Patients with Child-Pugh class A or those with Child-Pugh class B who were downstaged to class A through preoperative treatment; (4) postoperative pathological confirmation of HCC; (5) postoperative pathological confirmation of negative surgical margins. The exclusion criteria were: (1) patients undergoing a second surgery due to HCC recurrence; (2) patients with other malignancies or a history of related malignant tumors; (3) incomplete clinical or follow-up data; and (4) patients with a history of preoperative antitumor therapy. After preoperative downstaging therapy, we assessed the patient’s liver function recovery to Child-Pugh class A through the five parameters of the Child-Pugh score (bilirubin, albumin, INR, ascites, and hepatic encephalopathy), thereby confirming compliance with the indications for hepatic resection surgery. The specific inclusion and exclusion process is illustrated in **Figure 1**. Patients from Center 1 were randomly divided into a training set (n=221) and an internal validation set (n=96) in a 7:3 ratio, while Center 2 (n=58) served as the external validation set.

### 2.1 Types and standards of clinical data collection, related formulas, and definitions

The clinical data collected in this study consisted of the following variables: (1) General demographic information: Gender, age, and history of hepatitis B virus (HBV) infection; (2)Laboratory tests: Neutrophils (N), lymphocytes (L), platelets (PLT), aspartate aminotransferase (AST), alanine aminotransferase (ALT), gamma-glutamyl transferase (GGT), total bilirubin (TB), albumin (ALB), globulin (GLB), activated partial thromboplastin time (APTT), fibrinogen (FIB), and alpha-fetoprotein (AFP); (3) Tumor size; (4) History of cirrhosis; (5)Other relevant indicators: Gamma-glutamyl transferase-to-platelet ratio (GPR), albumin-to-fibrinogen ratio (AFR), aspartate aminotransferase-to-platelet ratio index (APRI), and changes in transaminases (CAA). Related calculation formulas: GPR = GGT/PLT; AFR = ALB/FIB; APRI = (AST/upper limit of normal AST/PLT) × 100. To better align with clinical decision-making and avoid potential uncertain effects of nonlinear relationships inherent in continuous variables on the model’s predictive performance, this study categorized clinical variables based on their normal reference ranges.

### 2.2 Construction of the composite variable CAA

The last preoperative (t_b_) and postoperative day 3 (POD3) values of aspartate aminotransferase (AST) and alanine aminotransferase (ALT) were recorded. The changes in AST and ALT were calculated based on the difference between the measurements on postoperative day 3 (POD3) and the last preoperative measurement. The difference value is defined as:


$${\text{ΔALT}}_{3}=\text{ALT}\left({\text{POD}}_{3}\right)-\text{ALT}\left({\text{t}}_{\text{b}}\right)$$

$${\text{ΔAST}}_{3}=\text{AST}\left({\text{POD}}_{3}\right)-\text{AST}\left({\text{t}}_{\text{b}}\right)$$

Since the numerical differences may span a magnitude range from 10^0^ to 10^3^ (which could potentially affect algorithm performance), the difference values were standardized using a scaling factor of 100:


$${\text{ΔALT}}_{3}=\frac{\text{ALT}\left({\text{POD}}_{3}\right)-\text{ALT}\left(t_{b}\right)}{100}$$

$${\text{ΔAST}}_{3}=\frac{\text{AST}\left({\text{POD}}_{3}\right)-\text{AST}\left(t_{b}\right)}{100}$$

The standardized ΔALT3 and ΔAST3 can be regarded as a two-dimensional vector, V = [ΔALT3, ΔAST3]. To quantify the magnitude of vector V, the Euclidean norm was employed, which intuitively represents the geometric distance from the origin to the endpoint of the vector. The final AST and ALT change index (CAA) was constructed using the following formula:


$$\text{ CAA}={\left\|\text{V}\right\|}_{2}=\sqrt{{\left({\text{ΔALT}}_{3}\right)}^{2}+{\text{ΔAST}}_{3}^{\;2}}$$

## 3 Data processing and analysis

Statistical analysis was performed using RStudio (version 4.3.3). Continuous variables conforming to a normal distribution were expressed as mean ± standard deviation (M ± SD) and compared using the t-test. Categorical variables were expressed as n (%) and compared using the chi-square test. Variable screening was conducted through univariate and multivariate logistic regression analyses. In the univariate analysis, *P* value <0.1was considered statistically significant, while in the multivariate analysis, *P* value <0.05was considered statistically significant. The logistic regression machine learning algorithm was used to construct the model. The “regplot” package (version 4.3.2) in RStudio was utilized to calculate and construct the nomogram. The “pROC” package (version 6.8-1) in RStudio was employed to plot the receiver operating characteristic curve (ROC), and the area under the curve (AUC) was used to evaluate the predictive performance of the nomogram. The “rms” package (version 6.8-1) in RStudio was used to construct calibration curves to assess the consistency between predicted probabilities and actual probabilities. The “ggDCA” package (version 1.1) in RStudio was applied to plot the decision curve analysis (DCA) to evaluate the clinical applicability of the model. Additionally, to enhance the understanding of the “black box” nature of the logistic regression machine learning model, the SHAP (SHapley Additive exPlanations) framework was employed for visual interpretation and analysis.

## 4 Results

### 4.1 General characteristics

The baseline data of the training set and internal validation set, randomly divided from 317 patients in Center 1, as well as the external validation set from Center 2, are shown in **Table 1**. As seen in **Table 1**, there were no significant statistical differences in clinical factors among the training set, internal validation set, and external validation set (P > 0.05).

**Table 1:** Baseline clinical characteristics of HCC patients.

Variables	Training (*n*=221)	Internal validation (*n*=96)	Externalvalidation (*n*=58)	*P* value
Sex,*n(%)*				0.886
Female	32 (14.5%)	14 (14.6%)	7 (12.1%)	
Male	189 (85.5%)	82 (85.4%)	51 (87.9%)	
Age,*n(%)*				0.088
≤50	78 (35.3%)	23 (24.0%)	15 (25.9%)	
>50	143 (64.7%)	73 (76.0%)	43 (74.1%)	
Size,(mean ± SD)	6.71 ± 3.94	6.58 ± 3.51	5.54± 3.65	0.112
GPR,(mean ± SD)	0.77 ± 1.06	0.88 ± 1.01	1.00± 1.36	0.327
AFR,(mean ± SD)	15.4 ± 5.19	14.9 ± 4.69	15.7 ± 8.03	0.626
APRI,(mean ± SD)	0.97 ± 1.13	0.84 ± 0.69	1.36 ± 2.35	0.058
ALBI,*n(%)*				0.854
1	117 (52.9%)	51 (53.1%)	33 (56.9%)	
2	102 (46.2%)	43 (44.8%)	25 (43.1%)	
3	2 (0.90%)	2 (2.08%)	0 (0.00%)	
CAA,*n(%)*				0.408
<5	126 (57.0%)	60 (62.5%)	38 (65.5%)	
≥5	95 (43.0%)	36 (37.5%)	20 (34.5%)	
HBV,*n(%)*				0.678
Negative	102 (46.2%)	48 (50.0%)	30 (51.7%)	
Positive	119 (53.8%)	48 (50.0%)	28 (48.3%)	
Cirrhosis,*n(%)*				0.538
Negative	52 (23.5%)	27 (28.1%)	17 (29.3%)	
Positive	169 (76.5%)	69 (71.9%)	41 (70.7%)	
N(×10^9^/L),*n(%)*				0.389
<1.8	24 (10.9%)	15 (15.6%)	5 (8.62%)	
1.8-6.3	188 (85.1%)	76 (79.2%)	48 (82.8%)	
>6.3	9 (4.07%)	5 (5.21%)	5 (8.62%)	
L(×10^9^/L),*n(%)*				0.423
<1.1	64 (29.0%)	21 (21.9%)	16 (27.6%)	
≥1.1	157 (71.0%)	75 (78.1%)	42 (72.4%)	
PLT(×10^9^/L),*n(%)*				0.9
>100	179 (81.0%)	79 (82.3%)	46 (79.3%)	
≤100	42 (19.0%)	17 (17.7%)	12 (20.7%)	
ALT(U/L),*n(%)*				0.082
≤50	177 (80.1%)	70 (72.9%)	39 (67.2%)	
>50	44 (19.9%)	26 (27.1%)	19 (32.8%)	
AST(U/L),*n(%)*				0.526
>40	96 (43.4%)	44 (45.8%)	30 (51.7%)	
≤40	125 (56.6%)	52 (54.2%)	28 (48.3%)	
TB(μmol/L),*n(%)*				0.514
≤21	160 (72.4%)	74 (77.1%)	40 (69.0%)	
>21	61 (27.6%)	22 (22.9%)	18 (31.0%)	
ALB(g/L),*n(%)*				0.74
≤40	108 (48.9%)	43 (44.8%)	26 (44.8%)	
>40	113 (51.1%)	53 (55.2%)	32 (55.2%)	
GLB(g/L),*n(%)*				0.51
≤35	192 (86.9%)	80 (83.3%)	52 (89.7%)	
>35	29 (13.1%)	16 (16.7%)	6 (10.3%)	
APTT(S),*n(%)*				0.488
<42	208 (94.1%)	87 (90.6%)	54 (93.1%)	
≥42	13 (5.88%)	9 (9.38%)	4 (6.90%)	
FIB(g/L),*n(%)*				0.739
≥2	186 (84.2%)	84 (87.5%)	49 (84.5%)	
<2	35 (15.8%)	12 (12.5%)	9 (15.5%)	
AFP(μg/L),*n(%)*				0.348
<400	175 (79.2%)	70 (72.9%)	42 (72.4%)	
≥400	46 (20.8%)	26 (27.1%)	16 (27.6%)	
GGT(U/L),*n(%)*				0.097
≤60	106 (48.0%)	37 (38.5%)	20 (34.5%)	
>60	115 (52.0%)	59 (61.5%)	38 (65.5%)	
ALTp(U/L),*n(%)*				0.922
≤50	7 (3.17%)	4 (4.17%)	2 (3.45%)	
>50	214 (96.8%)	92 (95.8%)	56 (96.6%)	
ASTp(U/L),*n(%)*				0.062
>40	218 (98.6%)	96 (100%)	55 (94.8%)	
≤40	3 (1.36%)	0 (0.00%)	3 (5.17%)	
GGTp(U/L),*n(%)*				0.642
≤60	115 (52.0%)	45 (46.9%)	31 (53.4%)	
>60	106 (48.0%)	51 (53.1%)	27 (46.6%)	
TBp(μmol/L),*n(%)*				0.314
≤21	119 (53.8%)	59 (61.5%)	29 (50.0%)	
>21	102 (46.2%)	37 (38.5%)	29 (50.0%)	
ALBp(g/L),*n(%)*				0.134
≤40	192 (86.9%)	87 (90.6%)	46 (79.3%)	
>40	29 (13.1%)	9 (9.38%)	12 (20.7%)	
GLBp(g/L),*n(%)*				0.213
≤35	218 (98.6%)	95 (99.0%)	57 (98.3%)	
>35	3 (1.36%)	1 (1.04%)	1 (1.72%)	

N, Neutrophils; L, Lymphocytes; PLT, Platelets; AST, Aspartate Aminotransferase; ALT, Alanine Aminotransferase; GGT, Gamma-Glutamyl Transferase; TB, Total Bilirubin; ALB, Albumin; GLB, Globulin; APTT, Activated Partial Thromboplastin Time; FIB, Fibrinogen; AFP, Alpha-Fetoprotein; GPR, Gamma-Glutamyl Transferase-to-Platelet Ratio; AFR, Albumin-to-Fibrinogen Ratio; APRI, Aspartate Aminotransferase-to-Platelet Ratio Index; ALBI, Albumin-Bilirubin Grade; CAA, Change in Transaminases; ASTp, Postoperative Aspartate Aminotransferase; ALTp, Postoperative Alanine Aminotransferase; GGTp, Postoperative Gamma-Glutamyl Transferase; TBp, Postoperative Total Bilirubin; ALBp, Postoperative Albumin; GLBp, Postoperative Globulin.

### 4.2 Analysis of independent factors influencing early recurrence after hepatectomy for HCC

Univariate analysis was used to evaluate the risk factors influencing early recurrence after hepatectomy for HCC. To include more variables for analysis, a cutoff value of P < 0.1 was set. Risk factors with P < 0.1 were further analyzed using multivariate regression to identify independent risk factors for early recurrence after hepatectomy for HCC. The results showed that tumor size, CAA, history of HBV infection, TB, AFP, and postoperative ALT were independent risk factors for early recurrence after hepatectomy for HCC (P < 0.05) (**Table 2**).

**Table 2:** Univariate and multivariate logistic regression analysis of early recurrence after hepatectomy for HCC in the training set.

Variables	Univariate			Multivariate		
	*OR* value	95*CI*	*P* value	*OR* value	95*CI*	*P* value
Size	1.03	1.02-1.05	<0.001	1.02	1.00-1.04	0.017
GPR	1.08	1.01-1.15	0.018	1.02	0.96-1.09	0.516
ALBI						
1						
2	1.04	0.91-1.19	0.524			
3	1.00	0.50-2.03	0.991			
CAA						
<5						
≥5	1.45	1.28-1.64	<0.001	1.42	1.25-1.60	<0.001
HBV						
Negative						
Positive	1.13	0.99-1.29	0.075	1.21	1.08-1.37	0.002
AST(U/L)						
>40						
≤40	0.86	0.75-0.98	0.021	0.94	0.82-1.08	0.389
AFP(μg/L)						
<400						
≥400	1.29	1.10-1.51	0.002	1.18	1.02-1.37	0.030
GGT(U/L)						
≤60						
>60	1.24	1.09-1.41	0.002	0.96	0.80-1.14	0.639
TB(μmol/L)						
≤21						
>21	1.19	1.02-1.37	0.023	1.15	1.00-1.31	0.047
ALTp(U/L)						
≤50						
>50	0.70	0.48-1.02	0.067	0.64	0.45-0.89	0.010
GGTp(U/L)						
≤60						
>60	1.18	1.04-1.35	0.012	1.10	0.94-1.29	0.240
ALBp(g/L)						
≤40						
>40	0.82	0.68-1.00	0.048	0.95	0.79-1.14	0.576

AST, Aspartate Aminotransferase; GGT, Gamma-Glutamyl Transferase; AFP, Alpha-fetoprotein; GPR, Gamma-glutamyl Transferase-to-Platelet Ratio; ALBI, Albumin-Bilirubin Grade; CAA, Change in Transaminases; TB, Total Bilirubin; ALTp, Postoperative Alanine Aminotransferase; GGTp, Postoperative Gamma-glutamyl Transferase; ALBp, Postoperative Albumin.

### 4.3 Model comparison and establishment

**Figure 2:**
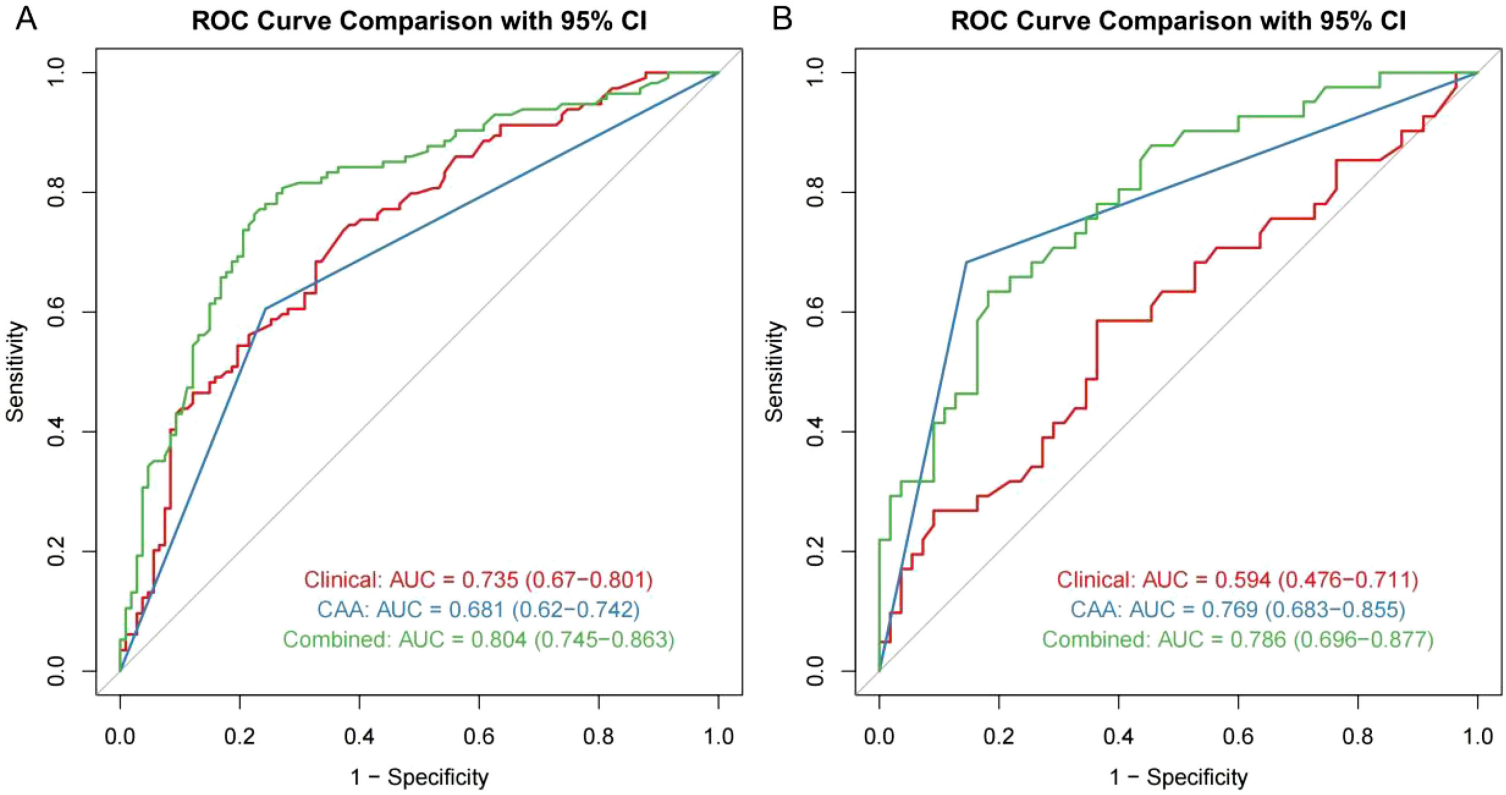
ROC model comparison. **(A, B)** ROC model comparison for the training and internal validation.

**Figure 3:**
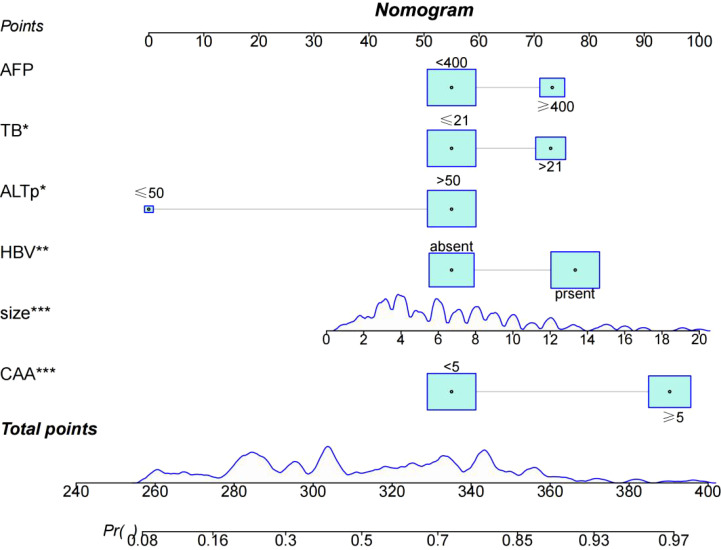
Nomogram for evaluating early recurrence after hepatectomy for hepatocellular carcinoma.

Through univariate and multivariate logistic regression analysis, independent risk factors including tumor size, CAA, history of HBV infection, TB, AFP, and postoperative ALT were identified. We compared CAA, other clinical variables, and their combination in the training set. The results showed that individual variables had poor predictive performance, while their combination significantly improved prediction accuracy (**Figure 2A**). This finding was further validated in the internal validation set, yielding consistent results (**Figure 2B**). Based on the model comparison results, we constructed a nomogram for the combined model to predict early recurrence after hepatectomy for hepatocellular carcinoma (**Figure 3**). The results indicated that CAA had the greatest impact, while the specific contributions of other factors are illustrated in **Figure 3**.

### 4.4 Visualization of the SHAP framework

**Figure 4:**
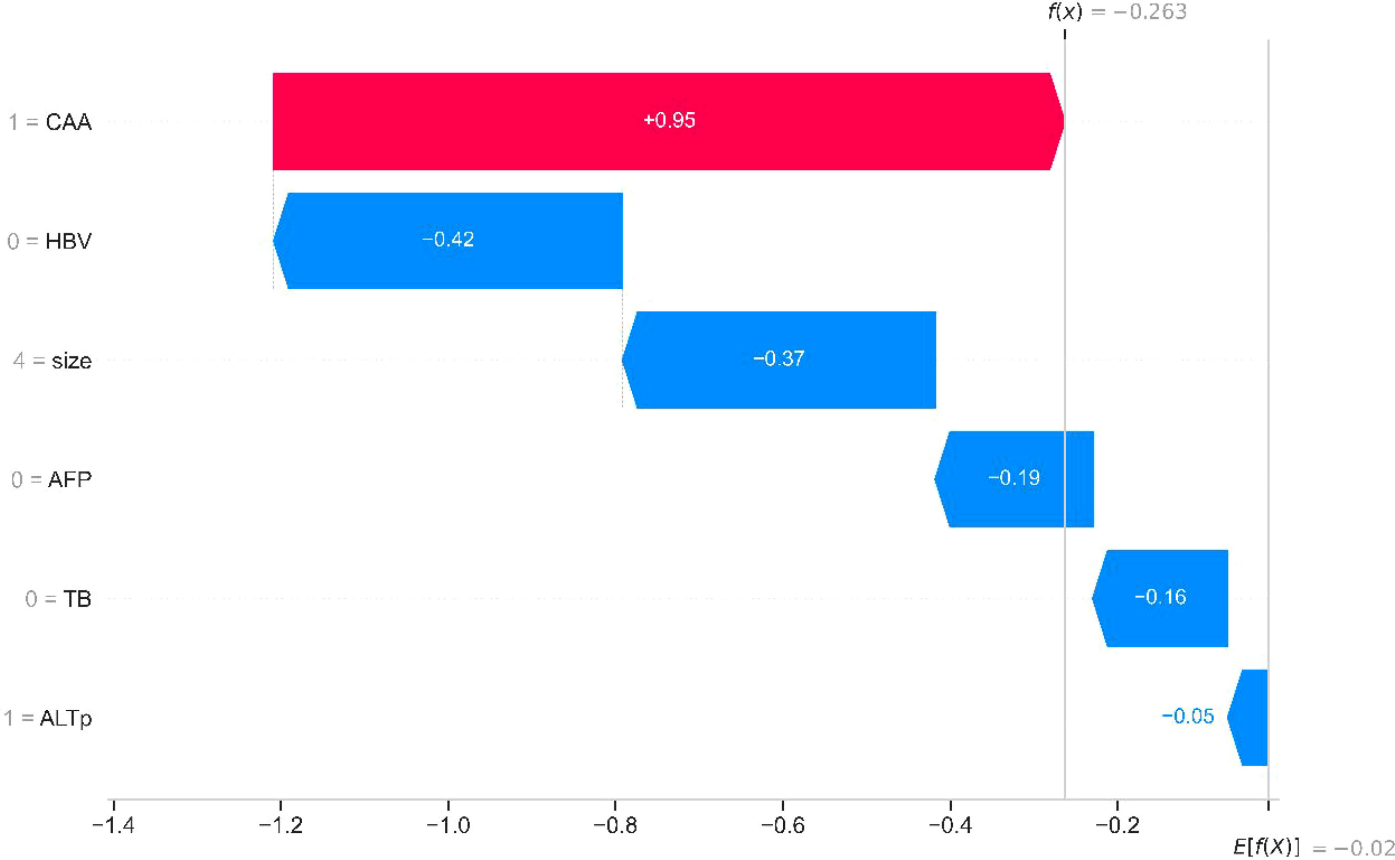
SHAP waterfall plot of risk factors in the internal validation set.

The SHAP method integrates multiple additive feature attribution techniques. By leveraging classical Shapley values and their extensions, SHAP establishes a connection between optimal credit allocation and local explanations, thereby enabling visualized interpretation of machine learning models. Building on this, researchers employed SHAP to elucidate the aforementioned models. **Figure 4** demonstrates the application of SHAP waterfall plots on an internal validation set, facilitating localized visualization (with independent risk factors delineated). Red denotes the positive contribution of high feature values to model predictions, while blue represents the negative impact of low feature values on predictions. As illustrated in the figure, the CAA indicator exerts the most significant influence on the model.

### 4.5 Validation of the predictive model

**Figure 5:**
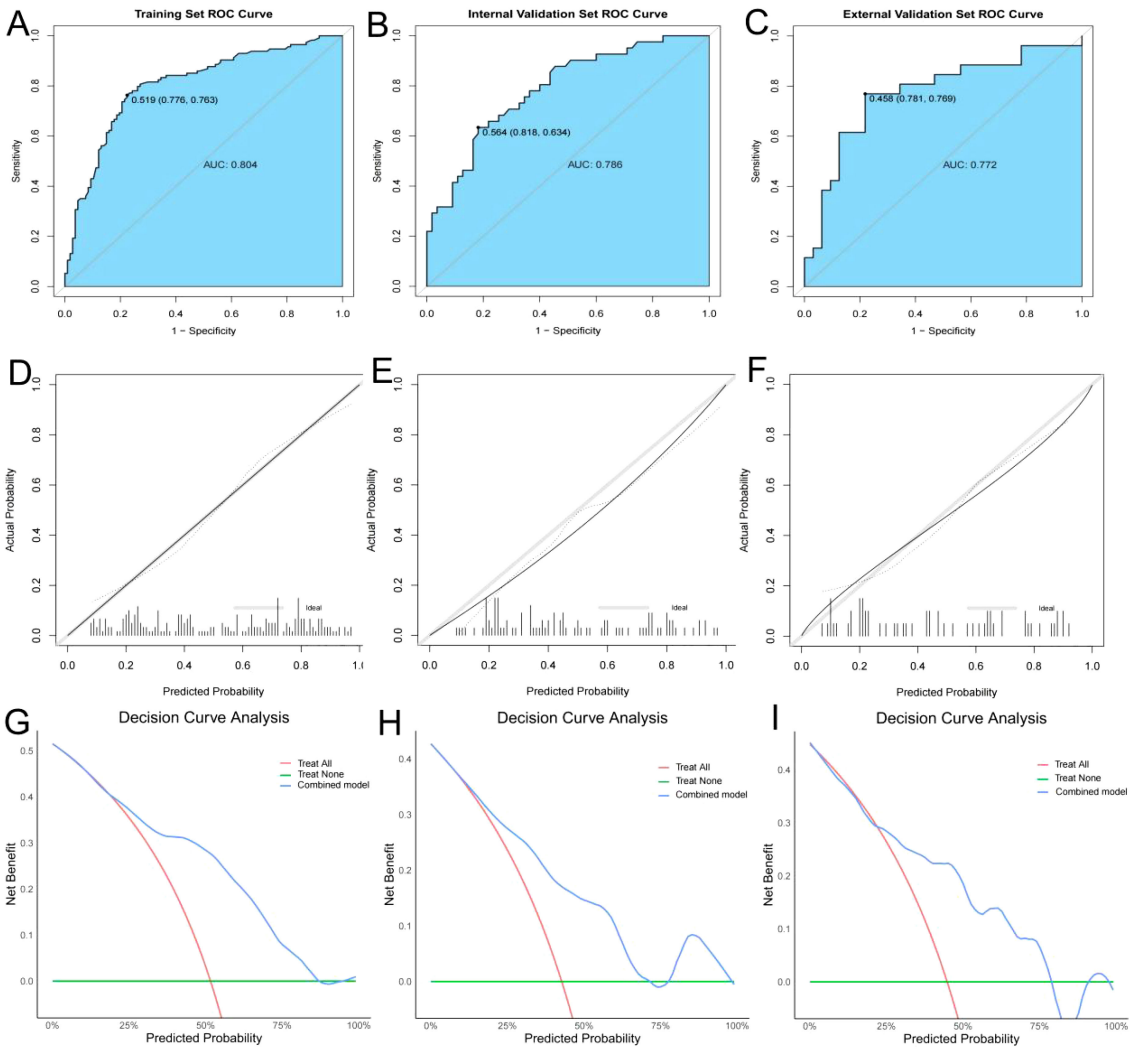
ROC curves, calibration curves, and DCA curves for the training, internal validation, and external validation. **(A–C)** ROC curves for the training, internal validation, and external validation; **(D–F)** Calibration curves for the training, internal validation, and external validation; **(G–I)** Decision curve analysis (DCA) curves for the training, internal validation, and external validation.

The predictive ability of the combined model was further validated by plotting ROC curves and calculating the AUC. The AUC values for the training set (**Figure 5A**), internal validation set (**Figure 5B**), and external validation set (**Figure 5C**) were 0.804, 0.786, and 0.772, respectively, indicating that the model has strong predictive performance. To further validate the calibration performance and clinical applicability of the model, calibration curves and decision curve analysis (DCA) curves were also plotted for the combined model. The calibration curves (**Figures 5D–F**) demonstrated that the nomogram has excellent clinical calibration ability. The DCA curves showed that the combined model has significant clinical benefits (**Figures 5G–I**).

### 4.6 Stratification of PFS and OS by the combined model

**Figure 6:**
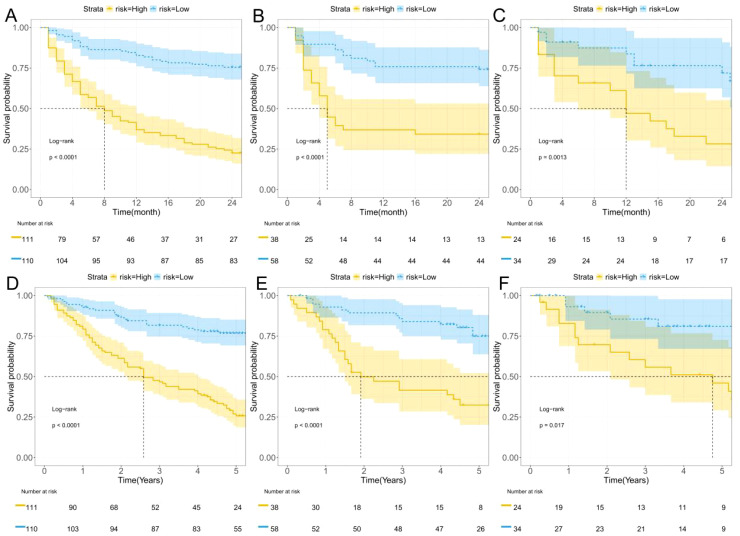
K-M curves. **(A–C)** Two-year progression-free survival (PFS) for the training, internal validation, and external validation; **(D–F)** Five-year overall survival (OS) for the training, internal validation, and external validation.

In this study, the maximum Youden index (0.519) from the training set of the combined model was used as the optimal cutoff value. This cutoff value was applied to the training set, internal validation set, and external validation set to stratify patients into low-risk and high-risk groups. The stratified patients were analyzed for 2-year progression-free survival (PFS) and 5-year overall survival (OS) using Kaplan-Meier curves. As shown in the figures, the predictive values of the combined model effectively stratified patients for 2-year PFS (**Figures 6A–C**) and 5-year OS (**Figures 6D–F**).

## 5 Discussion

Hepatocellular carcinoma (HCC) is the most common primary liver tumor and the fifth most prevalent malignant tumor worldwide (1). Surgical resection remains the primary treatment modality for HCC (17). However, early recurrence (typically defined as recurrence within 2 years postoperatively) after hepatectomy remains the most significant factor affecting long-term survival (4).In recent years, with advancements in laparoscopic techniques and the application of the Da Vinci robotic system, surgical trauma and postoperative complications have significantly decreased (18), and mortality rates have declined. However, the recurrence rate remains as high as 25%-50% (5). This study included 317 patients who underwent hepatectomy for HCC at the First Affiliated Hospital of the University of Science and Technology of China and 58 patients from the Third Affiliated Hospital of Anhui Medical University. Baseline characteristics showed no significant statistical differences. Univariate and multivariate logistic regression analyses were used to screen relevant variables. To include more variables, factors with a P-value < 0.1 in univariate analysis were included in multivariate analysis. This study identified tumor size, CAA, history of HBV infection, TB, AFP, and postoperative ALT as independent risk factors (P < 0.05) influencing early postoperative recurrence. We compared CAA, other clinical variables, and their combination, constructing a nomogram to predict early postoperative recurrence.

The study found that when the tumor diameter exceeds 5 cm, the probability of early postoperative recurrence is significantly higher than in patients with tumors smaller than 5 cm, consistent with multiple studies (19, 20). A tumor diameter greater than 5 cm is a high-risk factor for postoperative recurrence. Li et al. also noted that when the tumor diameter exceeds 5 cm, the probability of early recurrence increases significantly, and the mortality rate is 4.5 times higher than in patients with smaller tumors. When the tumor diameter exceeds 10 cm, the risk of extrahepatic recurrence also increases significantly (21). This may be related to microvascular invasion, as the incidence of microvascular invasion increases with tumor size. Further analysis suggests that, anatomically, the early recurrence of large tumors may be related to their anatomical relationships. Larger tumors are more likely to breach the Glisson sheath, leading to portal vein tumor thrombus (PVTT). Wang et al. pointed out that HCC associated with PVTT results in faster intrahepatic metastasis and liver dysfunction, leading to early recurrence and poorer prognosis. Additionally, at the molecular level, when the tumor size exceeds 5 cm, the hypoxic microenvironment and accumulation of inflammatory factors lead to the enrichment of CD133+ liver cancer cells, which is approximately 3.5 times higher than in patients with smaller tumors (22). Man KF et al. also noted that CD133+ liver cancer cells can maintain stemness through signaling pathways such as SPINK1 and Akt/PKB and actively expel chemotherapeutic drugs, leading to chemoresistance. Furthermore, CD133+ liver cancer cells enhance their invasiveness through epithelial-mesenchymal transition (EMT) and promote distant metastasis by secreting angiogenic factors such as VEGF (23), significantly increasing the risk of early recurrence.

In this study, the CAA index reflects the changes in serum transaminase levels during the perioperative period, including changes in postoperative ALT levels. Transaminases are the most direct indicators of liver function, and their changes not only reflect the degree of hepatocyte damage but also correlate closely with early recurrence after liver cancer surgery (24). In this study, we observed that when CAA ≥ 5, the probability of early recurrence increases significantly. Similarly, when postoperative ALT exceeds 50 U/L, the risk of recurrence also rises significantly. After hepatectomy, serum transaminase levels increase, likely due to the use of low central venous pressure and permissive hypotension during surgery, which inevitably leads to ischemia/reperfusion injury. Maspero et al. noted that the ischemic phase primarily manifests as mitochondrial dysfunction, with hepatocytes unable to function normally under hypoxic conditions, leading to cell death. After reperfusion, the release of reactive oxygen species (ROS) triggers microvascular dysfunction. In the later stages of ischemia/reperfusion injury (IRI), the immune system is activated, with massive infiltration of neutrophils, macrophages, and T cells, further exacerbating ROS production and hepatocyte damage (25). Guan et al. found that ROS production activates the Hippo-YAP pathway, promoting the proliferation of residual tumor cells and accelerating recurrence (26). Additionally, after hepatectomy, due to ischemia/reperfusion injury, liver sinusoidal endothelial cells (LSECs) undergo swelling and apoptosis due to oxidative stress and inflammatory factors such as TNF-α, leading to structural damage in the liver sinusoids. Damaged LSECs release vascular endothelial growth factor (VEGF) and angiopoietin-2 (ANGPT2), increasing vascular permeability and providing a physical pathway for circulating tumor cells (CTCs) to extravasate (27), thereby promoting intrahepatic metastasis and early recurrence.

In this study, we found that patients with a history of HBV infection had a significantly higher probability of early postoperative recurrence compared to those without HBV infection, consistent with the findings of Lu et al. (28)In patients with HBV infection, persistent antigen stimulation may induce PD-1 overexpression in CD8+ T cells, leading to T-cell exhaustion and weakening the immune response to pathogens and tumors (29). Additionally, in chronic liver disease, NKG2D function is inhibited, likely due to the chronic inflammatory microenvironment. NKG2D, an important activating receptor in the innate immune system, plays a crucial role in immune regulation against tumors, initiating immune surveillance and clearance in the early stages of tumor development. The downregulation of NKG2D function impairs immune surveillance and antitumor capabilities, leading to a state of “immune exhaustion” (30). When patients are infected with HBV, the direct carcinogenic effects of viral proteins may further influence early postoperative recurrence. Kgatle et al. noted that the HBV-related HBx protein upregulates DNA methyltransferases, leading to the suppression of tumor suppressor genes (31). When tumor suppressor genes are inhibited, the likelihood of tumor development increases significantly.

The study found that elevated total bilirubin (TB) levels often indicate metabolic dysfunction in hepatocytes. Bilirubin, a common oxidative stress substance, induces oxidative stress by increasing ROS levels. Oxidative stress dynamically regulates the expression of SLC7A11 (32), which reduces the generation of lipid peroxidation products, thereby decreasing the production of intracellular ferroptosis inducers and reducing ferroptosis. Consequently, upregulation of SLC7A11 significantly enhances tumor cell resistance to ferroptosis. This mechanism not only helps tumor cells survive under oxidative stress but may also affect the efficacy of tumor treatment (33). In this study, TB > 21 μmol/L was defined as elevated total bilirubin, which, although not causing visible jaundice, falls within the range of subclinical jaundice (21–34.2 μmol/L). This cutoff value can be used for early identification of potential bilirubin metabolism abnormalities. We found that when TB > 21 μmol/L, the risk of early postoperative recurrence increased significantly, consistent with the aforementioned research.

AFP, a commonly used tumor marker, is particularly significant in the diagnosis of liver cancer. Studies have shown that when AFP levels consistently exceed 400 ng/mL, liver cancer can be highly suspected (34). Recent research has found that AFP not only serves as a tumor marker but also promotes liver cancer recurrence by regulating the characteristics of liver cancer stem cells (35). Additionally, Ashokachakkaravarthy et al. discovered that liver cancer stem cells possess the ability to undergo mitotic dormancy, allowing them to remain quiescent during treatment and recur after therapy (36). The immune escape effect of AFP is another reason for the high early recurrence rate after liver cancer surgery. AFP inhibits immune responses and remodels the tumor microenvironment through various mechanisms, further suppressing antitumor immunity (37). Recent studies have found that AFP-L3, a glycosylated isoform of AFP, demonstrates higher clinical utility. Patients positive for AFP-L3 have a significantly increased risk of postoperative recurrence and are associated with poor tumor differentiation and vascular invasion (38). In this study, when AFP > 400 ng/mL, the risk of early postoperative recurrence was higher than in patients with AFP < 400 ng/mL, consistent with international research. AFP is not only a diagnostic marker in liver cancer but also a multifunctional molecule driving tumor progression. By regulating stem cell characteristics, immune escape, and remodeling the tumor microenvironment, AFP forms a pro-tumor network. Although targeting AFP therapy remains challenging due to tumor heterogeneity and drug resistance, the growing understanding of AFP-L3 and its prognostic value provides new directions for personalized treatment.

In this study, we introduced the new CAA index to construct a combined predictive model for early recurrence after hepatectomy for HCC, significantly improving predictive performance. Based on ROC model comparisons, in the training set, the AUC value was 0.735 when using other clinical indicators without CAA, 0.681 when using only CAA, and 0.804 when combining CAA with other indicators. To further validate these results, the internal validation set was analyzed, showing an AUC of 0.594 without CAA and 0.786 with CAA, consistent with the training set results. Based on the model comparison results, a nomogram for the combined model was constructed, with CAA having the greatest impact. Additionally, to visualize the interpretation of the combined model, SHAP plots were generated, indicating that CAA contributed the most to the prediction results. This suggests that higher CAA values are associated with a higher risk of early recurrence after hepatectomy. The SHAP plots also illustrate the contribution distribution of each feature, helping to better understand the factors influencing early recurrence. To further evaluate the reliability of the combined model, calibration curves were plotted. To assess the clinical utility and value of the combined model, decision curve analysis (DCA) was performed. The DCA curves demonstrated that the combined model has strong clinical decision-making capabilities, indicating that the model incorporating CAA exhibits superior predictive performance for early recurrence after hepatectomy.

To further analyze the survival time proportions of patients after treatment, Kaplan-Meier curves were plotted. The study found that the 2-year progression-free survival (PFS) of HCC patients after hepatectomy gradually declined over time, while the risk of recurrence increased. Using the maximum Youden index (0.519) as the optimal cutoff value, patients were divided into high-risk and low-risk groups. The high-risk group showed a faster decline in survival probability and a higher risk of recurrence compared to the low-risk group. Similarly, in the 5-year overall survival (OS) analysis, the high-risk group exhibited a faster decline in survival probability and a higher risk of recurrence. These findings were consistent in both the internal and external validation sets.

The current study has several limitations. For instance, this research only collected serological indicators while lacking surgical-related parameters. Additionally, the limited sample size and single-region design resulted in insufficient external validation due to the absence of data from high-incidence regions such as East Asia and sub-Saharan Africa. Although only patients with Child-Pugh class A were included, this category encompasses both scores of 5 and 6, which may reflect different levels of liver functional reserve. Due to the limited sample size, we did not perform a further subgroup analysis between these two scores. Future studies with larger cohorts are warranted to investigate potential differences within the class A group. Although patients initially classified as Child-Pugh B achieved restoration to Child-Pugh A after treatment, heterogeneity in hepatic functional reserve or residual portal hypertension may still exert a significant impact on clinical outcomes. Future studies should incorporate long-term follow-up and comprehensive multidimensional assessments of liver function (e.g., HVPG, ICG clearance, and imaging biomarkers) to further validate these findings. Furthermore, microvascular invasion might influence early recurrence risk, necessitating prospective studies incorporating pathological indicators to further validate the model’s efficacy. Therefore, future multicenter, large-scale, and prospective studies are warranted to validate and refine this model by integrating perioperative biochemical dynamics with intraoperative and pathological variables. Such comprehensive approaches may enhance early risk stratification and support more individualized postoperative surveillance strategies.

## 6 Conclusion

In conclusion, this study successfully constructed a predictive model for early recurrence after hepatectomy for HCC based on perioperative changes in serum transaminase levels. The model identified independent risk factors, including tumor size, CAA, history of HBV infection, TB, AFP, and postoperative ALT. Furthermore, this study highlights the importance of closely monitoring perioperative changes in serum transaminase levels to predict the likelihood of early postoperative recurrence. As research on early recurrence after hepatectomy for HCC continues to advance, it will provide critical support for improving patient prognosis and achieving personalized treatment.

## Data Availability

The raw data supporting the conclusions of this article will be made available by the authors, without undue reservation.
